# Supplementary material for: Aberrant integration of Hepatitis B virus DNA promotes major restructuring of human hepatocellular carcinoma genome architecture
Source: Nat Commun. 2021 Nov 25;12:6910. doi: 10.1038/s41467-021-26805-8 (PMC8617174; doi:10.1038/s41467-021-26805-8)
Supplement: Supplementary file 1 — Supplementary Info [file 41467_2021_26805_MOESM1_ESM.pdf]

SUPPLEMENTARY INFORMATION

Aberrant integration of Hepatitis B virus DNA promotes major restructuring of human hepatocellular carcinoma genome architecture

Eva G. Álvarez, Jonas Demeulemeester, Paula Otero, Clemency Jolly, Daniel García-Souto, Ana Pequeño, Jorge Zamora, Marta Tojo, Javier Temes, Adrian Baez-Ortega, Bernardo Rodríguez-Martín, Ana Oitaben, Alicia L. Bruzos, Mónica Martínez-Fernández, Kerstin Haase, Sonia Zumalave, Rosanna Abal, Jorge Rodríguez-Castro, Aitor Rodriguez-Casanova, Angel Diaz-Lagares, Yilong Li, Keiran Raine, Adam P. Butler, Iago Otero, Atsuhi Ono, Hiroshi Aikata, Kazuaki Chayama, Masaki Ueno, Shinya Hayami, Hiroki Yamaue, Kazuhiro Maejima, Miguel G. Blanco, Xavier Forns, Carmen Rivas, Juan Ruiz-Bañobre, Sofía Pérez-del-Pulgar, Raúl Torres-Ruiz, Sandra Rodríguez Perales, Urtzi Garaigorta, Peter J. Campbell, Hidewaki Nakagawa, Peter Van Loo & Jose M. C. Tubio

INDEX

	Page
Supplementary Table 1…………………………………………………………..	3
Supplementary Figure 1……………………………………………………………	4
Supplementary Figure 2……………………………………………………………	6
Supplementary Figure 3……………………………………………………………	8
Supplementary Figure 4……………………………………………………………	10
Supplementary Figure 5……………………………………………………………	11
Supplementary Figure 6……………………………………………………………	13
Supplementary Figure 7……………………………………………………………	14

The following documents are provided as separate files:

Supplementary Data 1. Counts of HBV insertion events per tumour

Supplementary Data 2. HBV insertions in human HCCs from the PCAWG dataset

Supplementary Data 3. Genomic features at HBV insertion sites

Supplementary Data 4. Clonal status of HBV insertions

Supplementary Data 5. Real-time timing of whole genome duplication events along with HBV insertions clonal states

Supplementary Table 1. Genomic annotation of ONT long reads supporting HBV-mediated rearrangements shown in the long-read plots. Column 1 shows sample code; Column 2 (Read) shows the name of the individual ONT read that supports the relevant HBV rearrangement; Column 3 (Length) shows the read size; Columns 4, 5 and 6 provide, for each read, the mapping coordinates onto the human hg19 genome assembly or the HBV genome, being column 4 (StartPos) the start position, column 5 (EndPos) the end position, and column 6 (Chr) the hg19 chromosome or HBV strain accession number in GenBank.

Sample	Read	Length	StartPos	EndPos	Chr
SA529830	c8248f35-5540-4826-9e9c-976f8968ea01	36845	2	28819	8
SA529830	c8248f35-5540-4826-9e9c-976f8968ea01	36845	29160	33691	NC_003977.2
SA529830	c8248f35-5540-4826-9e9c-976f8968ea01	36845	33701	36815	17
SA529726	000cba9b-420c-49d5-bfe0-b28c96ccebe3	51581	30	42982	X
SA529726	000cba9b-420c-49d5-bfe0-b28c96ccebe3	51581	43141	46064	NC_003977.2
SA529726	000cba9b-420c-49d5-bfe0-b28c96ccebe3	51581	46266	51562	3
SA501511	439aa81c-8495-4a3a-aaf0-95992575ca90	8379	21	4993	8
SA501511	439aa81c-8495-4a3a-aaf0-95992575ca90	8379	5115	6623	NC_003977.2
SA501511	439aa81c-8495-4a3a-aaf0-95992575ca90	8379	6681	8344	10
SA501511	ba6f0d12-0e2c-487c-99dc-ade5c4538497	26901	1730	26888	8
SA501511	ba6f0d12-0e2c-487c-99dc-ade5c4538497	26901	1092	1447	NC_003977.2
SA501511	ba6f0d12-0e2c-487c-99dc-ade5c4538497	26901	49	985	13
SA501511	65d86580-c441-4901-a0e4-b649a9358784	17118	60	8627	8
SA501511	65d86580-c441-4901-a0e4-b649a9358784	17118	8691	10845	NC_003977.2
SA501511	65d86580-c441-4901-a0e4-b649a9358784	17118	10861	17062	4
SA501453	dd58521b-0a9b-4550-a4c4-16ff26c30c60	52953	23141	52933	11
SA501453	dd58521b-0a9b-4550-a4c4-16ff26c30c60	52953	22539	23128	NC_003977.2
SA501453	dd58521b-0a9b-4550-a4c4-16ff26c30c60	52953	34	22511	19
SA501424	53874689-28b3-4d8f-9851-03335e6e86ab	24918	527	20311	1
SA501424	53874689-28b3-4d8f-9851-03335e6e86ab	24918	20325	23999	NC_003977.2
SA501424	53874689-28b3-4d8f-9851-03335e6e86ab	24918	24125	24884	11
SA529726	abcd9bf0-d02f-4a3c-8caf-864e9ab06ae3	14398	60	9032	4
SA529726	abcd9bf0-d02f-4a3c-8caf-864e9ab06ae3	14398	9348	12237	NC_003977.2
SA529726	abcd9bf0-d02f-4a3c-8caf-864e9ab06ae3	14398	12422	14376	7
SA501481	5b43bb59-f728-4aea-b299-65228a02385e	54394	36	31655	1
SA501481	5b43bb59-f728-4aea-b299-65228a02385e	54394	31883	34080	NC_003977.2
SA501481	5b43bb59-f728-4aea-b299-65228a02385e	54394	34315	54382	9

Supplementary Figure 1

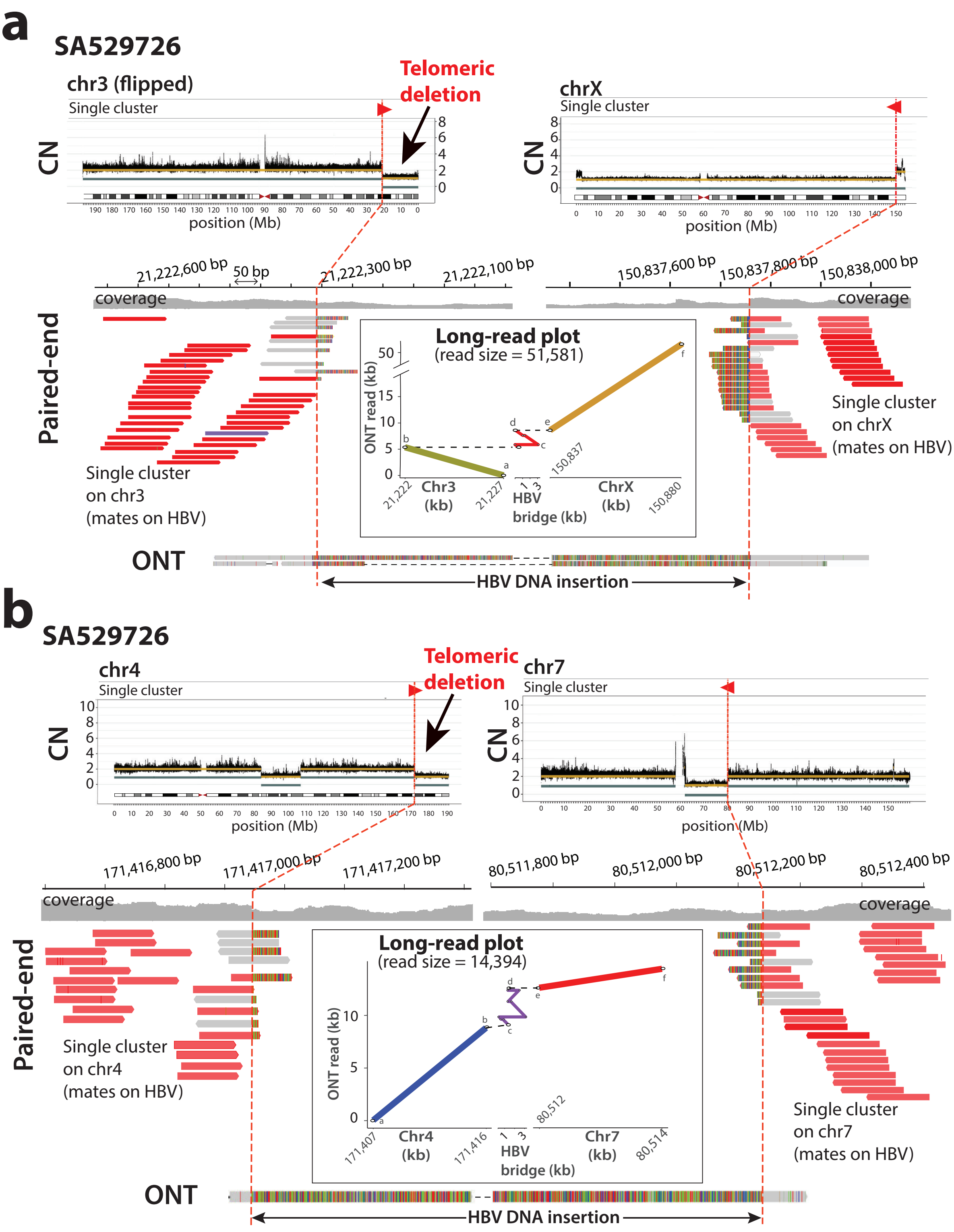

Supplementary Figure 1. HBV-mediated interchromosomal rearrangements revealed by ONT sequencing in HCC tumour SA529726. (a) The Illumina paired-end sequencing data (short-reads in red) shows one positive cluster of discordant read-pairs pointing to an HBV

insertion and demarcating the boundary of a 21.2 Mb telomeric deletion on 3p (note the orientation of the chromosome is inverted for illustrative purposes). The copy number plot (CN) at the top shows the total (gold line) and minor (grey line) chromosomes' copy number profiles. The ONT data at the bottom shows two long-reads obtained with Oxford Nanopore that reveals one 3,268 bp long HBV DNA insertion bridging the interchromosomal rearrangement between 3p and Xq. ONT reads were cut (discontinued) for illustrative purposes. A negative cluster of Illumina reads confirms the presence of the other extreme of the HBV insertion on Xq. The long-read plot represents the alignment of one ONT long-read to chromosomes 3 and X of the human reference genome and an HBV consensus sequence, which validates the interchromosomal rearrangement mediated by the virus. Note that the HBV insertion shows a typical rearranged pattern. **(b)** In the same sample, an HBV-mediated interchromosomal rearrangement between chromosomes 4 and 7 removes 19.8 Mb on 4q, including the telomere. Two paired-end clusters (short-reads in red) point to an HBV event. One ONT long-read reveals a 3,494 bp long HBV DNA insertion that bridges the translocation between 4q and 7q (long-read plot).

Supplementary Figure 2

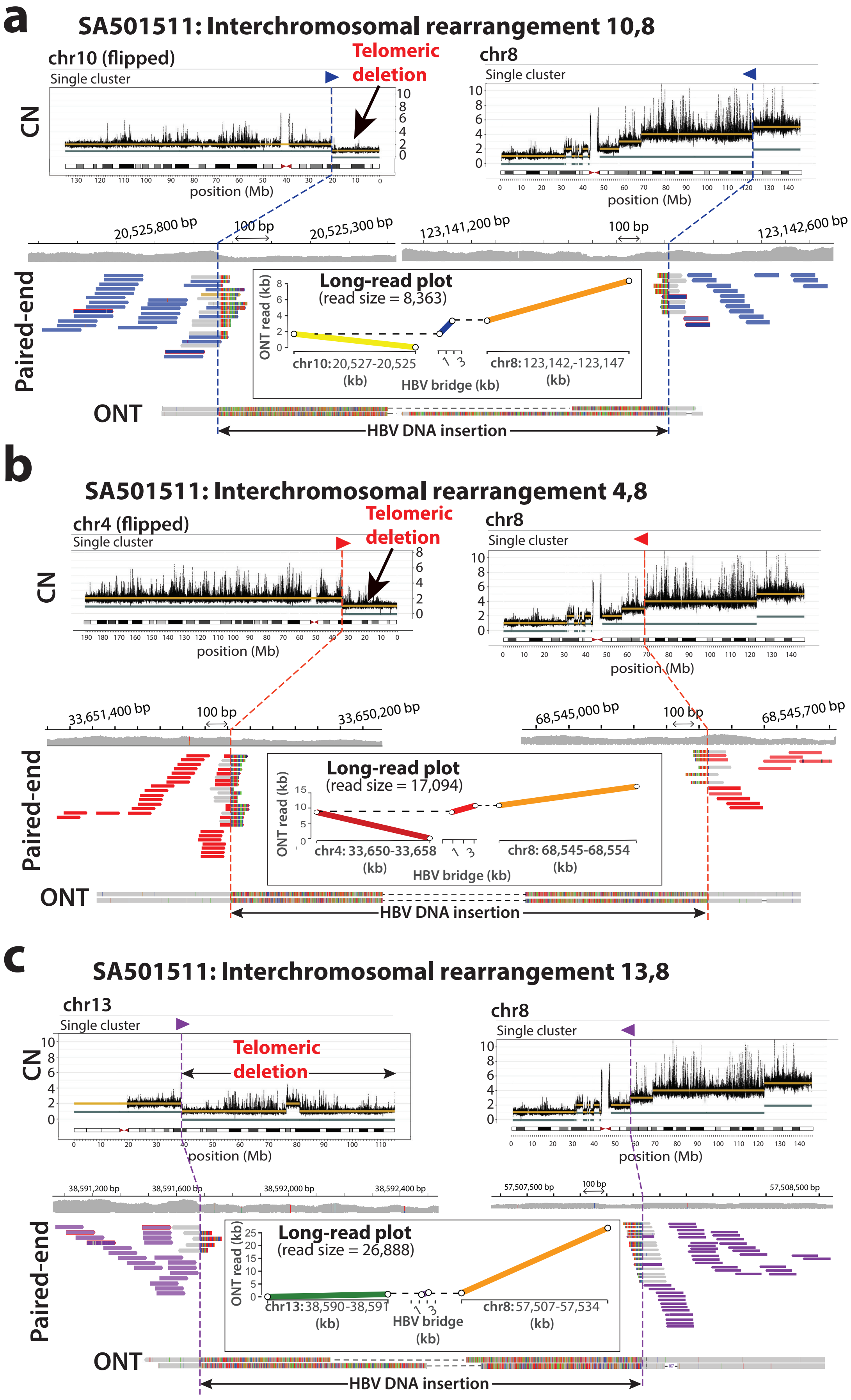

Supplementary Figure 2. HBV-mediated interchromosomal rearrangements revealed by ONT sequencing in HCC tumour SA501511. (a) The Illumina paired-end data (short-reads in blue) shows two clusters, one on chromosome 10 and another on chromosome 8, which point to both extremes of an HBV insertion. The copy number (CN) plot at the top shows the total (gold line) and minor (grey line) chromosomes' copy number profiles, revealing a 20.5 Mb telomeric deletion on 10p associated with the HBV insertion event (note that the CN plot on chromosome 10 is flipped for illustrative purposes). The ONT data at the bottom shows two long-reads that reveal the real configuration of the rearrangement. In the long-read plot, a 1,629 bp HBV insertion bridges an interchromosomal rearrangement between 10p and 8q. ONT reads were cut (discontinued) for illustrative purposes. **(b)** The Illumina paired-end data (short-reads in red) shows two clusters, one on chromosome 4 and another on chromosome 8, which point to both extremes of an HBV insertion. The CN plot at the top reveals a 33.6 Mb telomeric deletion on 4p associated with the HBV insertion event (note that the CN plot on chromosome 4 is flipped for illustrative purposes). The ONT data at the bottom shows two long-reads that reveal the real configuration of the rearrangement. In the long-read plot, a 2,227 bp HBV insertion bridges an interchromosomal rearrangement between 4p and 8q. **(c)** The Illumina paired-end data (short-reads in purple) shows two clusters, one on chromosome 13 and another on chromosome 8, which point to both extremes of an HBV insertion. The CN plot at the top reveals a 76.7 Mb deletion on 13q involving the telomere, which is associated with the HBV insertion event. The ONT data at the bottom shows two long-reads that reveal the real configuration of the rearrangement. In the long-read plot, a 531 bp HBV insertion that bridges an interchromosomal rearrangement between 13q and 8q.

Supplementary Figure 3

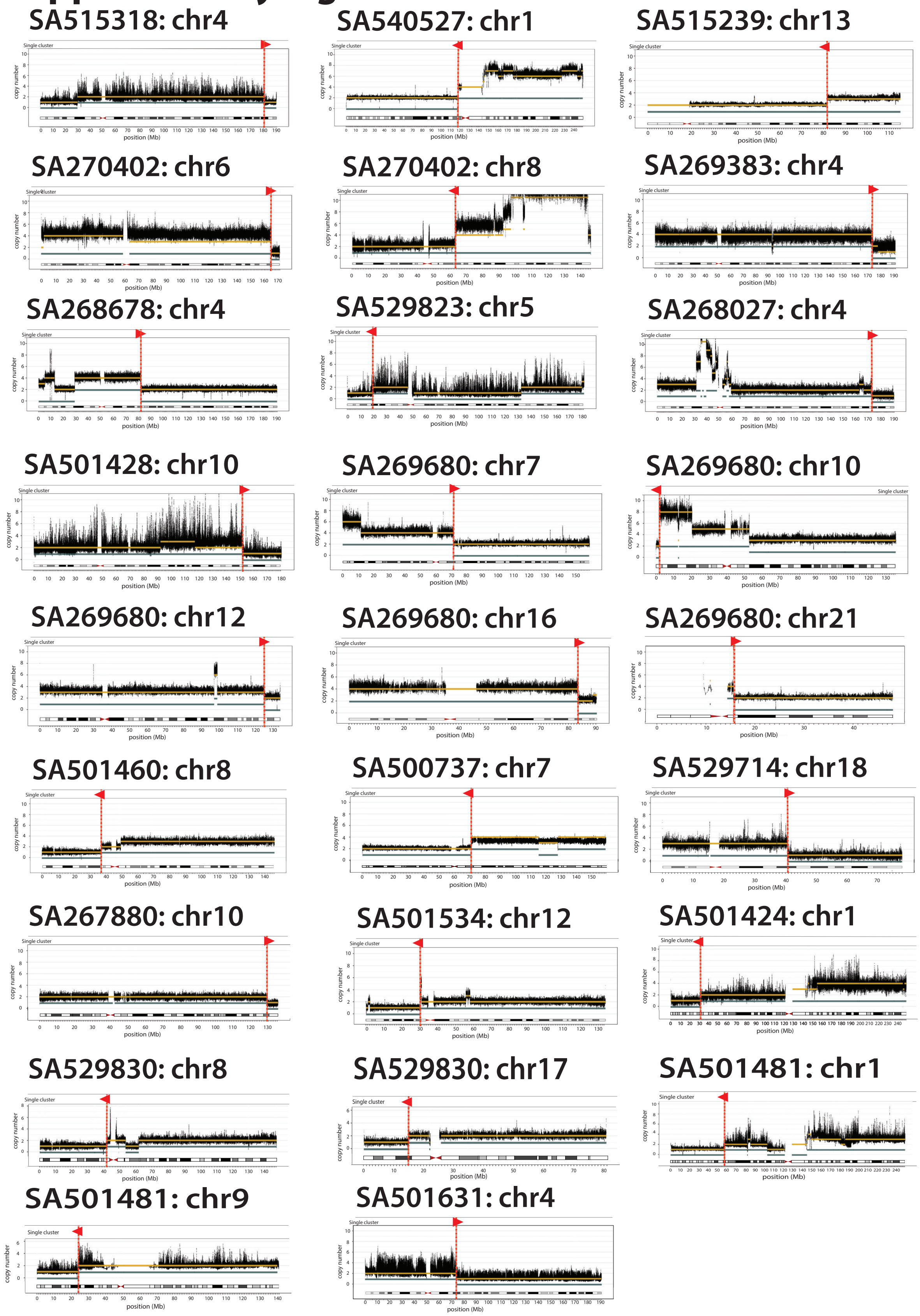

Supplementary Figure 3. Telomeric-like deletions associated with HBV DNA insertions are frequent in human HCC. Copy number (CN) plots from 26 chromosomes with telomeric-like deletions associated with HBV insertion events in HCC tumours from the PCAWG dataset. The CN plots show the total (gold line) and minor (grey line) chromosomes' copy number profiles. Arrows indicate the orientation (positive or negative) of the paired-end clusters supporting each HBV insertion. Note that a copy of the cancer-related gene *IRF2* is lost in chromosome 4 from samples SA269383 and SA268027.

Supplementary Figure 4

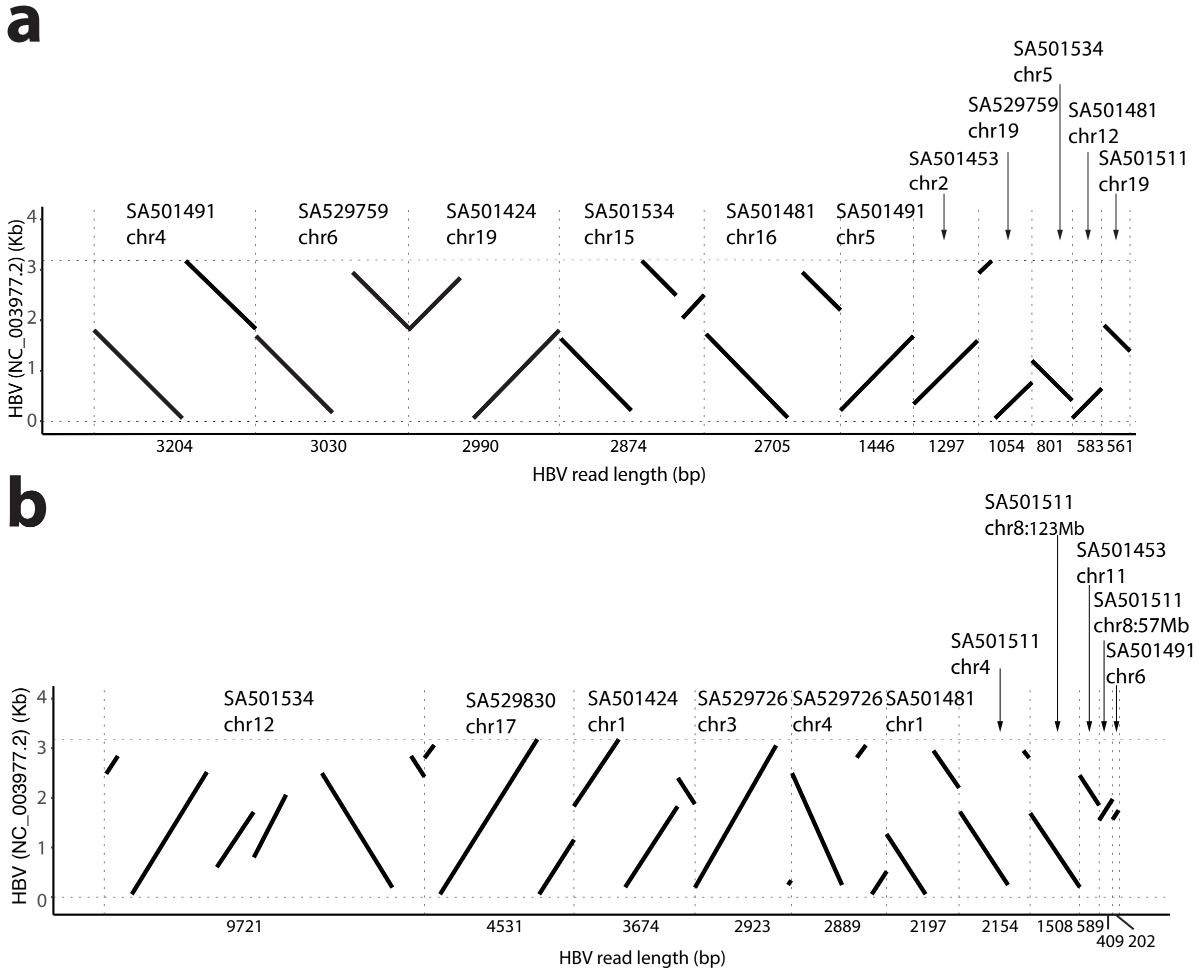

Supplementary Figure 4. Structural analysis of HBV insertions in HCC. Dot plot-like analysis of the HBV DNA sequences inserted in the HCC genome shows that HBV insertions at telomeric deletions tend to have a more complex structure than the HBV canonical insertions. Only those insertions sequenced with ONT and with full structure reconstructed are shown. The reference HBV sequence (NC_003977.2) is represented in the Y axis. Oxford Nanopore long-read length is displayed in the X axis. For each HBV insertion, the sample name and chromosome of insertion is shown. (a) HBV canonical insertions. (b) HBV non-canonical insertions linked to telomeric deletions.

Supplementary Figure 5

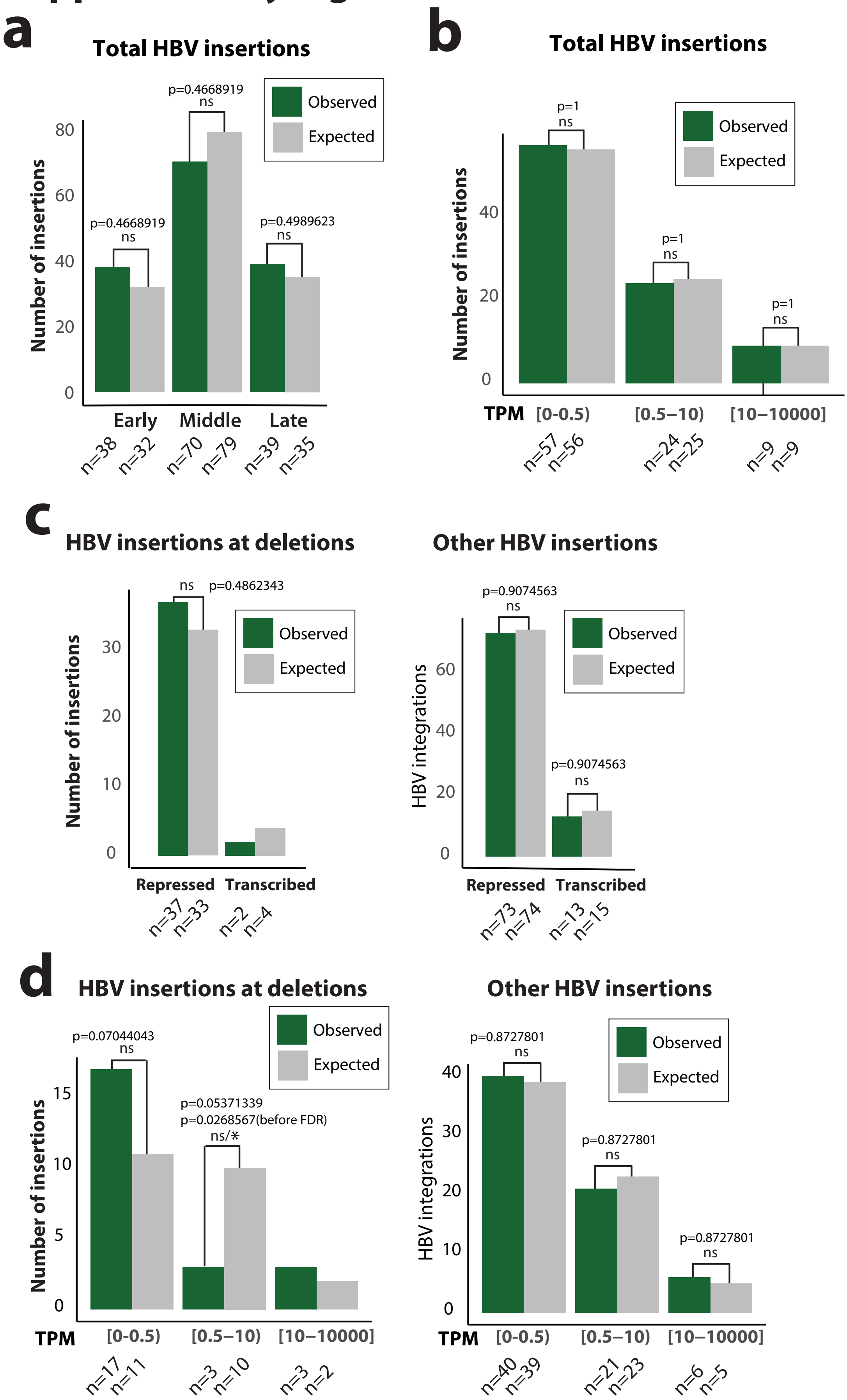

Supplementary Figure 5. Analysis of HBV insertion rate and genomic features. (a) Analysis of the frequency of total HBV insertions across replication timing categories (early, middle, late) showing no significant (ns) results. Chi-squared test and FDR correction were applied. **(b)** Same analysis for HBV insertion at genes with different expression levels. TPM denotes Transcripts Per Kilobase Million. No significant results are found after FDR correction. **(c)** Analysis of the frequency of total HBV insertions across chromatin states (Transcriptionally active and repressed chromatin), in HBV insertions at deletions (left) and the remaining HBV insertions (right), showing no significant results. Chi-squared test and FDR correction were applied. **(d)** Same analysis for HBV insertion at genes with different expression levels in TPM. No significant results are found after FDR correction. However, the HBV insertions at deletions were significantly more represented in the category of expression [0.5-10) before correction (p-value=0.02, n=3).

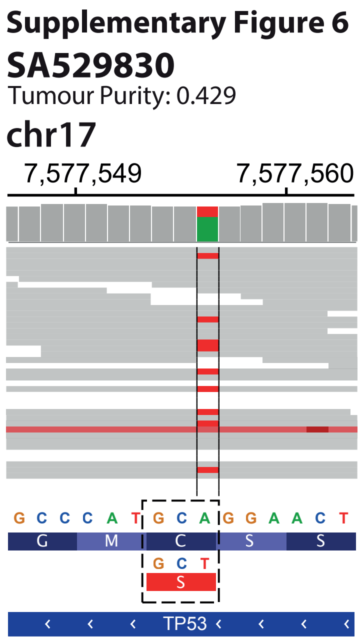

Supplementary Figure 6. Inactivating mutation in *TP53* from tumour SA529830. Inactivating mutation detected in the second allele of *TP53* from sample SA529830. The picture corresponds to an Integrative Genomics Browser (IGV) view of the paired-end mapping data at the relevant locus. An A>T mutation at exon 7 of the gene involves the aminoacid change C242S, catalogued as loss-of-function mutation [26]. Note that tumour purity is 0.429, which explains the reference ‘A’.

Supplementary Figure 7

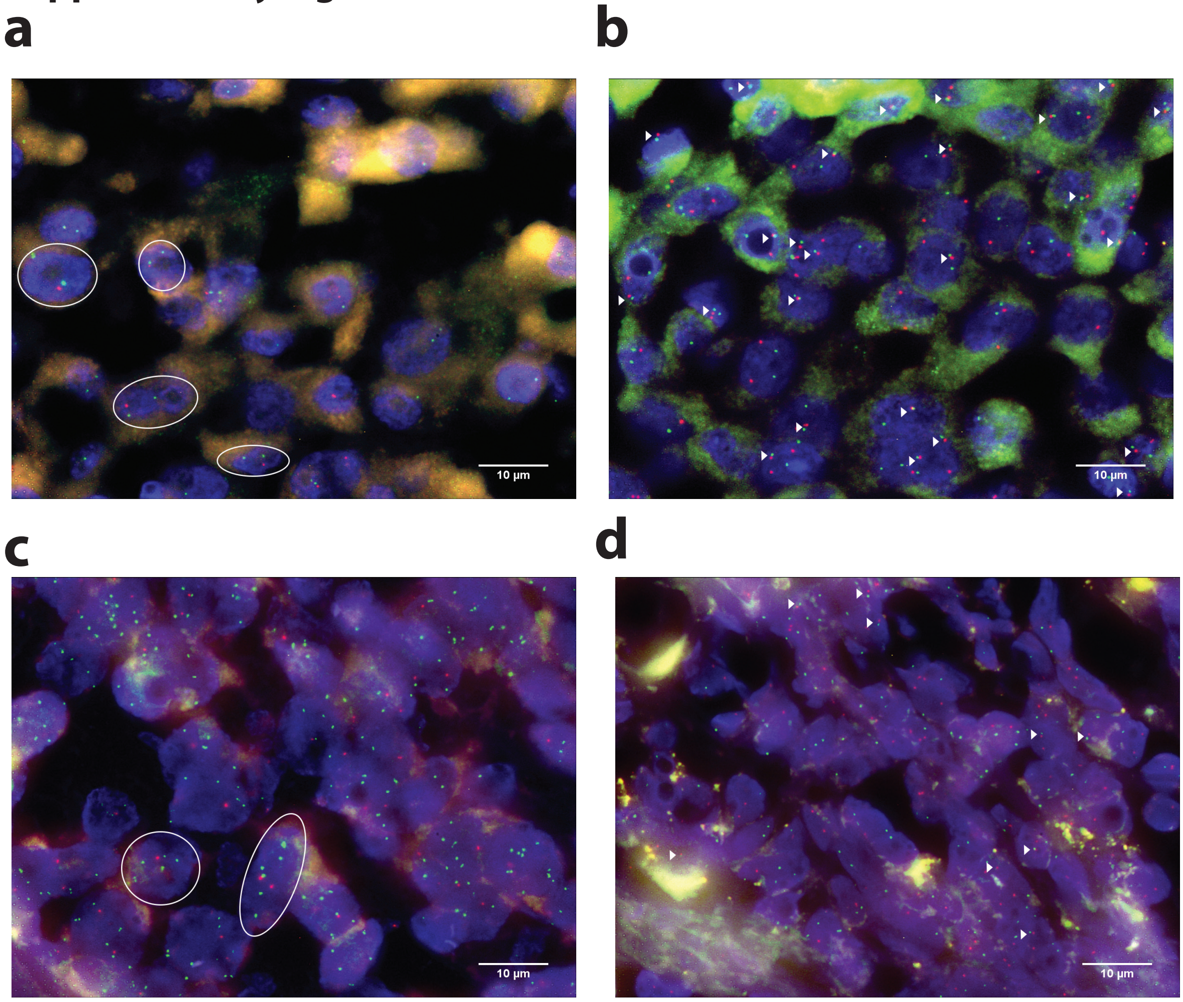

Supplementary Figure 7. Two colour fluorescence in-situ hybridization (FISH) analyses confirm driver reorganizations in HCC tumours. **(a)** FISH analysis of the TP53 deletion in HCC SA529830. Representative cells harbouring the deletion are shown inside the circles. Two green signals (control probe) and one red signal (TP53 probe) are detected in each cell. **(b)** Single-fusion FISH analysis of the t(8,17) translocation from the same tumour. In cells with the chromosomal fusion, one green and one red signal, each one targeting one the two chromosomes involved, colocalize together. In case of the standard (i.e., non-rearranged) karyotype, green and red signals split. Arrowheads point to the relevant chromosomal fusions. **(c)** FISH analysis of the ARID1A deletion in HCC SA501424. Representative cells harbouring

the deletion are shown inside the circles. Green signals (control probe) and red signals (ARID1A probe) are shown. In this case, between six to ten green signals and two red signals are detected in each cell, compatible with ARID1A deletion (see Methods). **(d)** Single-fusion FISH analysis of the t(1,11) translocation from the tumour SA501424. Arrowheads point to the relevant chromosomal fusions. Same slides from all tissue samples were scored by two independent observers.
